# The role of Insulin-like growth factor 2 mRNA-binding proteins (IGF2BPs) as m^6^A readers in cancer

**DOI:** 10.7150/ijbs.70458

**Published:** 2022-03-28

**Authors:** Chao-Yue Sun, Di Cao, Bin-Bin Du, Cun-Wu Chen, Dong Liu

**Affiliations:** 1 College of Biological and Pharmaceutical Engineering, West Anhui University, Lu'an, China.; 2 Department of Radiology, Sun Yat-sen University Cancer Center, State Key Laboratory of Oncology in South China, Collaborative Innovation Center for Cancer Medicine, Key Laboratory of Nasopharyngeal Carcinoma Diagnosis and Therapy, Guangzhou, 510060, P.R. China.

**Keywords:** N6-methyladenosine (m6A), IGF2BPs, Reader, Stabilization, Cancer

## Abstract

RNA can be modified by over 170 types of distinct chemical modifications, and the most abundant internal modification of mRNA in eukaryotes is N6-methyladenosine (m^6^A). The m^6^A modification accelerates mRNA process, including mRNA splicing, translation, transcript stability, export and decay. m^6^A RNA modification is installed by methyltransferase-like proteins (writers), and potentially removed by demethylases (erasers), and this process is recognized by m^6^A-binding proteins (readers). Notably, alterations of m^6^A-modified proteins (writers, erasers and readers) are involved in the tumorigenesis, progression and metastasis. Importantly, the fate of m^6^A-methylated mRNA is mediated mostly through m^6^A readers, and among these readers, insulin-like growth factor 2 mRNA-binding proteins (IGF2BPs) are unique RNA-binding proteins (RBPs) that stabilize their targets mRNA via m^6^A modification. In this review, we update the writers, erasers and readers, and their cross-talks in m^6^A modification, and briefly discuss the oncogenic role of IGF2BPs in cancer. Most importantly, we mainly review the up-to-date knowledges of IGF2BPs (IGF2BP1/2/3) as m^6^A readers in an m^6^A-modified manner in cancer progression.

## Introduction

The insulin-like growth factor 2 mRNA-binding proteins (IGF2BPs, IMPs), including IGF2BP1/2/3, are first identified in 1999 for their ability to bind to the name-giving 5'UTR of insulin-like growth factor Ⅱ (IGF-Ⅱ) leader 3 mRNA 1. The first IGF2BPs family member described is IGF2BP1 that is identified as a 75kd polysome-associated protein to stabilize c-MYC mRNA by binding to its coding region determinant (CRD) 2. IGF2BP2 is first described in 1999, and discovered as an intracellular antigen detected in 30-40% hepatocellular carcinoma patients 3, 4. IGF2BP3, initially called KOC protein, is first demonstrated as a highly expressed gene in pancreatic cancer that encodes four K-homologous (KH) domains 5. IGF2BPs are highly conserved RNA-binding proteins (RBPs) that are crucial players in regulating RNA processing, including mRNA splicing, translation, decay and stability 6. IGF2BPs are expressed in most tissues during embryogenesis, and only IGF2BP2 is ubiquitously expressed in adult tissues 3, 7. Importantly, IGF2BPs are characterized as oncofetal proteins, by which IGF2BPs are involved in carcinogenesis 8, 9. Accumulating data have been demonstrated that IGF2BPs are highly expressed in a broad range of tumors and also associated with poor prognosis 8.

IGF2BPs uniquely contain two RNA recognition motifs (RRMs) and four KH domains. KH domains-containing proteins are first reported by Kiledjian to regulate mRNA stability in 1995 10. The underlying mechanism of mRNA stabilization by KH domains is revealed by Huang laboratory, by which IGF2BPs stabilize c-MYC by its KH domains (KH3-4) in m^6^A modification manner 11. Since then, the function of IGF2BPs in m^6^A modification has been continuously reported, where the IGF2BPs participate in posttranscriptional process, including alternative splicing, metabolism, and stabilization 12.

In a manner similar with DNA and protein, RNAs can be modified by more than 170 chemically modifications 13. Among them, N6-methyladenosine (m^6^A) is most abundant modification of messenger RNA (mRNAs) and non-coding RNA (ncRNAs) in mammals 14, 15. Although first discovered in the 1970s, the m^6^A modification began to revive in 2012, when a next-generation sequencing method, MeRIP-Seq was described and used 16. In mammalian cells, m^6^A modification can be catalyzed by a methyltransferase complex (“writers”) and removed by demethylases (“erasers”), thus m^6^A mRNA modification is a reversible and dynamic process 17. Notably, the fate of m^6^A-modified RNA is mediated mostly through RNA-binding proteins (“readers”), including YTHDF, FMRP, CNBP, PRRC2A, HNRNPA2B1 and IGF2BPs 18-20. The m^6^A modification by m^6^A readers affects mRNA fate by regulating RNA splicing, translation, stability, structure, export and decay of the modified RNA 21. Recently, emerging evidence demonstrates that m^6^A modification also affects the mRNA fate by promoting the phase separation of m^6^A readers 22. For example, m^6^A regulates the fate of cytosolic mRNA through scaffolding for binding YTHDF proteins, resulting in the formation of phase separation complex (YTHDF-m^6^A-mRNA) that partitions into phase-separated compartments, such as P-bodies and stress granules 23. This effect is efficient for the polymethylated mRNAs that scaffold multiple YTHDF proteins. In addition, m^6^A modification is required for the liquid-liquid phase separation (LLPS) of YTHDC1 to form nuclear condensates that protect mRNAs from degradation, and regulate myeloid leukemic differentiation 24. In addition to m6A readers, the roles of LLPS in m^6^A writers have been explored, and for instance, LLPS is an important player in regulating dynamic assembly of the mRNA m^6^A methyltransferase complex (METTL3/METTL14/WTAP) 25.

m^6^A modification governs mRNA fate and function in multiple biological processes, and m^6^A readers-mediated m^6^A modification is implicated in various human diseases, especially cancer 26. Among m^6^A readers, IGF2BPs are very uniquely RNA-binding proteins that play critical roles in cancer progression through affecting mRNA fate in an m^6^A-dependent manner. In this review, we will briefly introduce the understanding of m^6^A modification by the writers, erasers and readers, and crosstalk between theses regulators. In addition, we also summarize the function of oncogenes, IGF2BPs in the tumorigenesis and cancer progression. Of note, we focus on how IGF2BPs promote tumorigenesis and progression via the m^6^A-dependent manner. Moreover, we further describe perspectives toward future questions and challenges in IGF2BPs-mediated m^6^A modification.

## m^6^A writers, erasers, and readers

m^6^A, one of the most abundant chemical modifications in eukaryotic mRNA, has gained increasing attention as a mode of post-transcriptional gene regulation 27-29. Genetic loss-of-function studies on m^6^A writers, erasers and readers highlight m^6^A modification as a dynamically and reversibly regulatory process in various biological processes 30, 31. m^6^A methylation plays critical roles in regulating gene expression through mediating the mRNA stability, degradation and translation, and its disruption results in a series of diseases, including cancer 32-34. The cross-talks among m^6^A writer, eraser, and reader are reported to determine the m^6^A levels of their targets and, consequently, the stability of these targets plays important roles in tumorigenesis, drug resistance, and metastasis 35-37.

### m^6^A writers

m^6^A is established by m^6^A methyltransferases complex (also called “writers”) that transfers a methyl group from s-adenosylmethionine (SAM) to the substrate adenosine in RNA 38, 39. Methyltransferase-like protein 3 (METTL3) and METTL14 play as a catalytic core complex known as the m^6^A-METTL complex (MAC) that recognizes the DRACH motifs and promotes the m^6^A modification in the transcriptome 40-42. Notably, METTL3 has catalytic activity, while METTL14 forms a heterodimer with METTL3 and then strengthens its catalytic action 39, 43, 44. Interestingly, MAC interacts with m^6^A-METTL associated complex (MACOM) that composes of the wilms tumor 1 associated protein (WTAP), vir-like m^6^A methyltransferase-associated (VIRMA), RNA-binding motif 15 (RBM15), zinc-finger CCCH-type-containing 13 (ZC3H13), Cbl proto oncogene-like protein 1 (CBLL1) 45. Although the MACOM itself lacks catalytic activity, its coordinated interaction with the MAC promotes it localization to nuclear speckles and modulates their recruitment to specific targets for m^6^A modification. METTL16 (a homologue of METTL3), a novel independent RNA methyltransferase, is a conserved U6 snRNA methyltransferase and regulates cellular SAM levels 46, 47. CAPAM (also known as PCIF1) has been recently identified as an evolutionarily conserved methyltransferase, responsible for the m^6^A on the mRNA cap-adjacent Am-modified nucleotide 48-50. The METTL5: TRMT112 complex has been recognized as a m^6^A rRNA methyltransferase that catalyzes the N6-methylation of A1832 (m^6^A1832) in human 18S rRNA 51. In addition, ZCCHC4, another m^6^A rRNA MTase, facilitates the m^6^A4220 modification in all human 28S rRNA 52.

### m^6^A erasers

The possible reversibility and dynamic of the m^6^A modification are identified by the m^6^A demethylases (erasers): fat mass and obesity-associated protein (FTO) and α-ketoglutarate-dependent dioxygenase alk B homolog 5 (ALKBH5) protein, which can selectively remove m^6^A-methylated groups from their targets RNA 53, 54. The demethylase activity of ALKBH5 has been demonstrated to be preferential for m^6^A methylation in DRACH motif-dependent manner in RNA, whereas FTO demethylate a wide array of substrates including m^6^A 54. Several recent studies suggest that FTO, however, preferentially demethylates the N6,2′-O-dimethyladenosine (m^6^Am), which suggests that ALKBH5 is more participated in global m^6^A demethylation than FTO 55.

### m^6^A readers

m^6^A recognition proteins, known as “readers”, can decode m^6^A marks and perform diverse biological functions 56, and these m^6^A readers include YT521-B homology domain family proteins (YTHDF1/2/3), YT521-B homology domain containing 1 and 2 (YTHDC1/2) 57, eukaryotic translation initiation factor 3 (eIF3) 58, insulin like growth factor 2 mRNA-binding proteins (IGF2BP1/2/3) 11, heterogeneous nuclear ribonucleoproteins (HNRNPA2/B1, HNRNPC/G) 59, Proline rich coiled-coil 2 A (Prrc2a) 60, HuR (known as ELAVL1) 41, cellular nucleic acid binding protein (CNBP) 61, and SND1 62. YTHDF2 is first identified m^6^A reader that impairs the stability of targeted transcripts and promotes mRNAs degradation via recruiting the CCR4-NOT deadenylation complex 57, 63. Conversely, YTHDF1 promotes mRNA translation by interacting with the translation initiation factor eIF3 64. YTHDF3 not only can interact cooperatively with YTHDF2 to promote mRNA decay, but also cooperates with YTHDF1 to promote the translation of the methylated RNAs 65, 66. Interestingly, YTHDF3 loss decreases the binding of YTHDF1 and YTHDF2 to their transcripts, displaying the important role of YTHDF3 in the RNA binding specificity 65. YTHDC1 mediates alternative splicing by recruiting the splicing factor serine and arginine-rich splicing factor 3 (SRSF3) and restricting the bind of exon-skipping factor SRSF10 67. YTHDC2 preferentially binds to m^6^A-containing RNA, and then promotes translation efficacy of its targets and reduce their mRNA abundance 68, 69. eIF3 can directly bind to mRNAs-containing m^6^A in their 5'UTR, which is sufficient to stimulate the mRNA translation in the loss of the cap-binding factor eIF4E 58.

The HNRNPs family binds to m^6^A-containing mRNA through the mechanism of “m^6^A-switch”, in which m^6^A modification alters the mRNAs structures to expose the single stranded hnRNP binding motif 59, 70. HNRNPA2B1 binds to pre-microRNA and accelerates its maturation 19, 71. In addition, HNRNPA2B1 also functions as modulating the alternative splicing of transcripts 19. HNRNPC participates in the processing of pre-mRNAs and intronless mRNAs 72. HNRNPG selectively binds to RNAs and regulates alternative splicing by interacting with m^6^A-methylated mRNAs 73.

Recently, IGF2BPs are verified as a distinct and conserved family of m^6^A-readers, consisting of two RNA recognition motif (RRM) domains and four K homology (KH) domains 7. Nevertheless, the third and fourth KH domains (KH3-4) are responsible for recognizing the m^6^A sites of targets mRNAs. IGF2BPs can promote the stability and facilitate the translation efficiency in an m^6^A-dependent fashion 11. And the IGF2BPs-mediated mRNA stabilization can be enhanced by recruiting the co-factors of IGF2BPs, HuR and matrin 3 (MATR3), to prevent their targets from degradation 11. In addition, IGF2BPs are also demonstrated to be involved in mRNA translation by modulating alternative splicing 74.

**Figure 1:**
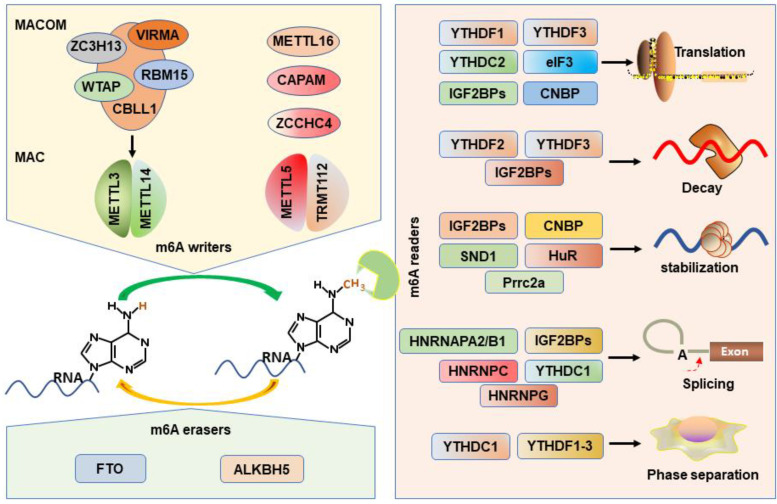
Working model of m^6^A writers, erasers and readers on m^6^A modification.

Prrc2a encodes a large proline-rich protein and is located at the MHC class III region 75. A recent study has proved that Prrc2a functions as a novel m^6^A reader stabilizing the mRNA of Olig2 60. HuR, one of the members of the Hu family of RNA-binding proteins, is associated with the m^6^A bait and stabilizes m^6^A-containing mRNAs 41, 76. In addition, the “Royal family” protein, SND1 also can read the m^6^A-modified mRNAs and promotes mRNA stabilization 62. CNBP is recently identified a novel m^6^A reader that consists of seven highly conserved zinc finger domains involved in mRNA transcription, stabilization, and translation 20, 61, 77. Interestingly, only CNBP protein is localized in the nucleoplasm while other readers are localized to the cytosol. However, all m^6^A writers are localized in the nucleus and most readers are localized to the cytosol, how writers-readers systems work remains poorly understood. The m^6^A writers, erasers, and readers have been characterized in **Figure 1**.

## Crosstalk between m^6^A writers, erasers, and readers

First, the links between m^6^A writers and readers have been extensively studied. m^6^A readers are often required for m^6^A methyltransferases-catalyzed m^6^A methylation. For example, METTL3-dependent m^6^A hypermethylation up-regulates NLRP1 transcript, and knockdown of YTHDF1 reduces the NLRP1 mRNA 78. In turn, m^6^A writers are also necessary for m^6^A readers-mediated m^6^A modification. For example, METTL14 depletion reduces Socs1 m^6^A methylation, and blunts YTHDF1 binds to the m^6^A sites 79. Loss of METTL3 impairs YTHDF1-mediated translation of its target, SPRED2 in an m^6^A modification manner 80. In addition, m^6^A writers and readers bind to the same target transcripts 81, and thus, m^6^A writers and readers combine together to regulate m^6^A methylation process. One reasonable possibility is that m^6^A writers and readers can form polymeric methyltransferase complex. Second, the relationships between the m^6^A erasers and readers are similar to the above links. m^6^A readers are also required for m^6^A demethylases-modified m^6^A methylation. For example, ALKBH5 suppresses pancreatic cancer progression through activation of PER1 in an YTHDF2-dependent m^6^A way 82. Third, m^6^A writers and erasers regulate m^6^A modification in opposite direction. METTL3 and ALKBH5, for example, oppositely regulate the TFEB mRNA level in an m^6^A-modified manner 83. In addition, m^6^A writers and erasers constitute positive feedback loops to control the stability of target transcripts 35. Fourth, m^6^A writers and erasers can determine the m^6^A status of targets by controlling each other's expression and regulating m^6^A readers 35. Thus, interplay among m6A writer, eraser and reader determines the m^6^A status and level.

## The oncogenic role of IGF2BPs in cancer

All human IGF2BPs have been identified as oncofetal proteins, and among them, IGF2BP1 and IGF2BP3 are *bona fide* oncofetal proteins that are synthesized in many cancers 3. In agreement, IGF2BP1 and IGF2BP3 display a high degree of amino acid identity (73%) with each other and show similar activity 84. The oncogenic role of IGF2BP3 was first described due to its overexpression in pancreatic cancer in 1997 5, and then IGF2BP3 modulates tumor cell fate, such as proliferation, migration, and chemo-resistance by controlling the translation and turnover of target transcripts, and regulating DNA methylation, and acetylation processes 3, 85. IGF2BP1 is the most conserved oncogene of all three IGF2BPs that is required for the transport, stability, and localization of mRNAs in carcinogenesis, and chemo-resistance 84, 86. Of note, IGF2BP1 is post-transcriptional driver of E2F1-driven hallmark in solid cancers 87. IGF2BP2 is a unique member of IGF2BPs that is ubiquitously expressed in the adult organism, and IGF2BP2 is an important post-transcriptional regulator of RNAs via the ribonucleoprotein complex 7, 8. Interestingly, IGF2BP2 can preferentially regulate glucose and lipid metabolism, and thus IGF2BP2 is considered as diabetes associated gene that impairs insulin secretion 88. Notably, accumulating data demonstrates that IGF2BP2 promotes carcinogenesis by regulating cancer metabolism 8.

Nonetheless, all three IGF2BPs are highly expressed and function as independent prognostic factors in a variety of cancers, including lung cancer 89, 90, liver cancer 91-93, breast cancer 94, 95, colorectal cancer 96-98, pancreatic cancer 99-101, prostate cancer 102, bladder cancer 103-105, thyroid cancer 106-108, gastric cancer 109, 110, renal cell carcinoma 111, ovarian cancer 112, 113, esophageal squamous cell carcinoma 114, 115, acute myeloid leukemia 116. In addition to the high expression of the IGF2BPs itself, intercellular communication factors, such as tumor-secreted extracellular vesicles (EVs), can maintain the stability of IGF2BPs in cells to promote the development of cancer. EVs are emerging as crucial messengers maintaining homeostasis in tumor progression, and metastasis 117. Interestingly, the RBPs and their substrate RNAs are detected in EVs, and EVs can harbour sequence motifs to mirror the activity of RBPs 118. circNEIL3 is packaged into exosomes and then transmitted to tumor associated macrophages (TAMs) that enable them to acquire immunosuppressive effect by stabilizing IGF2BP3, and promoting glioma progression 119. EVs secreted by melanoma cells regulate the effect of IGF2BP1 on metastasis, and in turn, IGF2BP1 affects the cargo of the EVs 120. Nevertheless, the connection of IGF2BPs' stability with EVs biology requires comparative and systematic studies. In addition, IGF2BPs also play important roles in regulating other cancer phenotypes, such as glycolysis, stemness and chemo-resistance 121-123. In mechanism, the oncogenic roles of IGF2BPs are largely attributed to their m^6^A dependent mRNA stabilization of oncogenic targets 87. Thus, in most cases, inhibition of IGF2BPs or their targets can suppress the proliferation and migration of cancer cells.

## The role of IGF2BPs as m^6^A reader in cancer

The m^6^A modification is a dynamic process, and the biological function of m^6^A relies on m^6^A readers. These readers recognize m^6^A-containing RNAs either by directly binding to YT521-B homology (YTH) domain, or by binding to single-stranded RNA motifs 124. Although YTH domains bind to RNA with low affinity between 100 and 300 nM, YTH domain-containing proteins recognize m^6^A to regulate mRNA splicing, metabolism, folding, and translation 17, 125. Besides YTH domain, m^6^A-containing RNAs can be selectively recognized by K homology (KH) domain. In 2018, IGF2BPs are first recognized as a novel family of m^6^A readers that stabilize their targets in an m^6^A-dependent manner 11. IGF2BPs have six characteristic RNA-binding domains, including four distinct KH domains at C-terminal region, and two RNA recognition motifs (RRMs) at N-terminal region 126. IGF2BPs-recognized m^6^A by KH domains has been demonstrated by Huang's laboratory, by which mutations in the KH domains (KH3-4) completely abolish the function of IGF2BPs as m^6^A reader 11. Importantly, the critical role of KH3-4 domains in cancer has been demonstrated before IGF2BPs are identified as new m^6^A reader 127. Unlike the canonical YTH domain-containing proteins, IGF2BPs-recognized targets, such as c-MYC, display higher transcript level and longer half-life period 124. As RNA stabilizers, IGF2BPs promote the stability of multiple mRNAs through m^6^A modification and IGF2BPs-modified m^6^A plays a crucial role in many pathological conditions, especially cancer.

### IGF2BP1

Currently, m^6^A modification regulates the translation 128, splicing 129, maturation 130, stabilization 131, and decay 132 of noncoding RNAs, including miRNAs, lncRNAs and circRNAs in cancer. In turn, interestingly, noncoding RNAs regulate IGF2BPs mRNA stabilization or IGF2BPs-modificated m^6^A in cancer progression 133, 134. LncRNA THAP7-AS1 is correlated with poor prognosis in gastric cancer patients, and mechanistically, METTL3 stabilizes THAP7-AS1 in IGF2BP1-mediated m^6^A modification manner 135. The oncogene MYC (also known as c-MYC) is one of the most frequently activated in human cancers, and c-MYC overexpression causes tumorigenesis and maintains tumor growth 136. Importantly, c-MYC is considered as a critical target of IGF2BPs, and lncRNAs recruits or binds to IGF2BP1 to stabilize or increase the mRNA of c-MYC, depending m^6^A modification in tumor progression. For example, a hypoxia-induced lncRNA KB-1980E6.3 maintains stemness of breast cancer stem cells (BCSC) under hypoxic microenvironment by recruiting IFG2BP1 to stabilize c-MYC mRNA in m^6^A-modified manner 137. Before identified as the m^6^A reader, IGF2BPs are regulated by lncRNAs to mediate translation and mRNA stability of c-MYC 138. Interestingly, lncRNAs regulate m^6^A modification on their targets' mRNA by IGF2BP1. LINC002661 encodes a 71-amino acid oncopeptide that binds to IGF2BP1 and then strengthens the m^6^A recognition of IGF2BP1 to increase mRNA stability of m^6^A-methylated c-MYC, which promotes tumorigenesis 139. In addition to lncRNAs, a study reported that circPTPRA plays as a tumor suppressor in bladder cancer by interacting the KH domains of IGF2BP1 to block its m^6^A recognition of its targets, c-MYC and FSCN1 mRNA 105.

In addition to noncoding RNAs, m^6^A modification by IGF2BP1 also regulates the function and generation of mRNAs. High expression of IGF2BP1 is associated with poor prognosis in endometrial cancer patients, and mechanistically, IGF2BP1 recruits polyadenylate-binding protein 1 (PABPC1) to stabilize paternally expressed gene 10 (PEG10) mRNA in an m^6^A-dependent manner 140. In endometrial cancer, IGF2BP1 is a direct downstream target of peptidylarginine deiminase II (PADI2) that is required for endometrial cancer progression, and IGF2BP1 binds to the m^6^A sites of oncogenic SOX2 and prevents its mRNA degradation 141. In hepatocellular carcinoma (HCC), ALKBH5, an m^6^A demethylase, inhibits LY6/PLAUR Domain Containing 1 (LYPD1) that is recognized and then stabilized by IGF2BP1 142. In HCC, RNA-binding motif protein 15 (RBM15) facilitates cancer progression via promoting post-transcriptional activation of YES1 in IGF2BP1-mediated m^6^A manner 143. In addition, ALKBH5 also blocks pancreatic cancer progression through activation of PER1 by another m^6^A reader, YTHDF2 82. In liver cancer stem cells (LCSC), IGF2BP1 facilitates LCSC phenotypes via promoting the stability of MGAT5 mRNA in an m^6^A modification manner 144. In another study, IGF2BP1 is demonstrated as a post-transcriptional enhancer of serum response factor (SRF) in cancer with a 3' UTR and m^6^A-dependent manner 145. A later study also shows that IGF2BP1 promotes tumorigenesis and metastasis of oral squamous cell carcinoma via enhancing Bmi1 mRNA translation in METTL3-mediated m^6^A modification manner 146. A subsequent study shows that METTL3 promotes the mRNA stability of kinesin-like protein, KIP3C in IGF2BP1- modified m^6^A manner, accelerating prostate cancer progression 147. Moreover, METTL3 methylates KRT7-AS to enhance the mRNA stability of keratin 7 (KRT7) depending on IGF2BP1-modified m^6^A, promoting breast cancer lung metastasis 148. In addition to m^6^A methyltransferases, m^6^A demethylase FTO reduce the stability of DACT1 mRNA by IGF2BP1-modified m^6^A demethylation, facilitating osteosarcoma progression 149. Recently, IGF2BP1 is reported to promote E2F1-3-driven G1/S transition in an m^6^A-dependent manner in cancer cells 87. Thus, IGF2BP1, as an m6A-reader, plays as an important oncogene in cancer by stabilizing or enhancing mRNA of its oncogenic factors, and thus IFG2BP1 is druggable for cancer treatment. Interestingly, m^6^A readers (IGF2BPs) cooperatively interact with m^6^A writers or erasers to regulate cancer progression.

### IGF2BP2

In similar with IGF2BP1, noncoding RNAs also interact with IGF2BP2 to stabilize or increase their targets mRNA, and IGF2BP2 can directly regulate noncoding RNAs in an m^6^A-dependent way. In colorectal cancer patients, high expressed LINC00460 is correlated with poor overall survival, and mechanistically, LINC00460 interacts with IGF2BP2 to bind to the 3'UTR of high-mobility group AT-hook 1 (HMGA1), and enhances the stability of HMGA1 mRNA 150. In colorectal liver metastasis model, circNSUN2 binds to the KH3-4 domains of IGF2BP2 and stabilizes the high mobility group AT-hook 2 (HMGA2) 151. In esophageal squamous cell carcinoma (ESCC), LncRNA CCAT2 increases IGF2BP2 expression, and then IGF2BP2 improves mRNA stability of thymidine kinase 1 (TK1) in m^6^A modification manner, which promotes tumor progression 152. In prostate cancer, lncRNA PCAT6 interacts with IGF2BP2 to promote bone metastasis by stabilizing insulin-like growth factor1 receptor (IGF1R) mRNA 153. In glioblastoma, IGF2BP2 recognizes the m^6^A site of lncRNA CASC9 and increases its stability, and CASC9 cooperates with IGF2BP2 to form a complex that stabilizes hexokinase 2 (HK2), promoting aerobic glycolysis 154. In pancreatic cancer patients, IGF2BP2 is associated with poorer prognosis, and mechanistically, IGF2BP2 plays as m^6^A reader for modification of lncRNA DANCR and stabilizes its mRNA 155. In thyroid cancer, lncRNA HAGLR increases IGF2BP2 expression, and IGF2BP2 recognizes the m^6^A modification of c-MYC and leads to increased c-MYC expression, which promotes cancer progression 156. In addition, lncRNA LINRIS stabilizes IGF2BP2 by blocking its ubiquitination to promote the c-MYC-driven glycolysis in colorectal cancer 157. In colorectal cancer, IGF2BP2 directly binds to the m^6^A sites of lncRNA ZFAS1 and increases its stability to activate the Warburg effect 158. Moreover, LINC01021 promotes tumorigenesis and progression through enhancing the mRNA stability of target transcripts, MSX1 and JARID2 in IGF2BP2- mediated m^6^A modification 159. Besides lncRNAs, circCD44 also directly interacts with IGF2BP2 to enhance the stability of c-MYC mRNA in m^6^A-modifed manner, promoting cancer progression in triple-negative breast cancer 160. In addition, in cervical cancer, circARHGAP12 interacts with IGF2BP2 through m^6^A site in the exon-3 to increase the stability of forkhead box M1 (FOXM1) mRNA 161. In turn, IGF2BP2 also directly stabilizes noncoding RNAs to promote cancer progression. For example, IGF2BP2 binds to lncRNA DUXAP9 and increases its stability in m^6^A manner, which facilitates proliferation and motility of renal cancer cells 162.

In addition to noncoding RNAs, the mechanism of IGF2BP2 activity also relies on the direct interaction with its protein partners. For example, higher expression of IGF2BP2 is associated with a poorer prognosis in HCC patients, and mechanistically, IGF2BP2 directly recognizes and binds to the m^6^A site of flap endonuclease-1 (FEN1), and maintains its mRNA expression 92. Analogously, in colorectal cancer, IGF2BP2 recognizes and binds to m^6^A-modified YAP and enhances the stability of YAP mRNA, thereby facilitating tumorigenesis 163. Similarly, IGF2BP2 recognizes the coding sequence (CDS) regions of transcription factor SOX2 and protects it from degradation in m^6^A- mediated manner, which facilitates tumorigenesis and metastasis in colorectal cancer 164. In similar with c-MYC, oncogene SOX2 is highly susceptible to m^6^A modification 165, 166. In thyroid cancer, m^6^A demethylase FTO inhibits cell growth and glycolysis by reducing the mRNA stability of target, APOE in IGF2BP2-mediated m^6^A-dependent manner 167. IGF2BP2 also promotes lymphatic metastasis and epithelial-mesenchymal transition (EMT) of head and neck squamous carcinoma cells by stabilizing slug mRNA in an m^6^A-dependent manner 168. In addition, human papillomavirus E6/E7 promotes aerobic glycolysis of cervical cancer by stabilizing MYC expression in IGF2BP2-mediated m^6^A-dependent way 169. In breast cancer stem-like cells (BCSC), aurora kinase A (AURKA) binds to IGF2BP2 and strengthens IGF2BP2 to stabilize DROSHA mRNA in an m^6^A-modified way, thereby increasing BCSC stemness maintenance 170.

In addition to cancer, IGF2BP2-mediated m^6^A modification also plays crucial roles in other physiological and pathological contexts. Most recently, IGF2BP2 maintains mitochondrial homeostasis of hematopoietic stem cells (HSCs) through maintaining the mRNA stability of its downstream target Bmi1, indicating that IGF2BP2-mediated m^6^A modification is critical for HSCs maintenance and hematopoiesis 171. In the immune process, IGF2BP2 regulates macrophage phenotypic activation by stabilizing TSC1 and PPARγ mRNA in an m^6^A-dependent manner 172. Moreover, IGF2P2 also binds to CCAAT/enhancer binding proteins (C/EBPs) to enhance the mRNA half-life and expression of C/EBPs in an m^6^A-modified manner in autoimmune inflammation 173. In addition, IGF2BP2-modified m^6^A also plays important roles in regulating cardiac hypertrophy and aging-associated disorders 174, 175.

### IGF2BP3

A major function of IGF2BP3 is interaction with the mRNA machinery, and plays as a stabilizer of oncogene in cancer 3. In colon cancer, high expression of IGF2BP3 is associated with poorer overall survival, and IGF2BP3 recognizes and binds to the CDS region of Cyclin D1 to regulate cycle, and IGF2BP3 also regulates angiogenesis through m^6^A modification of vascular endothelial growth factor (VEGF) 176. In gastric cancer, IGF2BP3 directly binds to hypoxia inducible factor-1a (HIF1A) at a specific m^6^A site in the CDS region, and knockout of IGF2BP3 inhibits cell migration and angiogenesis induced by hypoxia 177. In addition, IGF2BP3 can bind to the m^6^A-modified region of the ATP-binding cassette transporters subfamily B member 1 (ABCB1) and promotes its mRNA stabilization, thereby triggering chemoresistance of colorectal cancer cells 178. m^6^A demethylase ALKBH5 inhibits metastasis of gastric cancer through modulating expression of downstream target, PKMYT, and IGF2BP3 stabilize the mRNA stability of PKMYT1 by recognizing its m^6^A modification sites 179. m^6^A methyltransferase METTL3 post-transcriptionally mediates PD-L1 mRNA activation in breast cancer with m^6^A-IGF2BP3-dependent manner 180. Thus, m^6^A readers play important roles in m^6^A writers or erasers-mediated the stability of targets mRNA. Moreover, MYC-activated IGF2BP3 increase m^6^A-modified level of KPNA2, thereby promoting cell proliferation and metastasis nasopharyngeal carcinoma 181. Alternatively, IGF2BPs also can stabilize mRNAs in an m^6^A-independent manner. For example, IGF2BP3 specifically binds to pregenomic RNA (pgRNA) and increases its stability without m^6^A modification, and promotes the stemness and tumorigenicity of HCC cells 182. Thus, in addition to m6A modification, IGF2BPs can stabilize their targets mRNA through other mechanisms, and in general, preferentially through the m^6^A-modified manner.

In line with IGF2BP1 and IGF2BP2, noncoding RNAs play as guide or scaffold to recruit or interact with IGF2BP3 to regulate the function of their targets. For instance, lncRNA DMDRMR binds and cooperates with IGF2BP3 to stabilize multiple targets, including CDK4, in an m^6^A-dependent manner, and thereby drives cancer progression of clear cell renal cell carcinoma 111. In addition, circular RNA, circ-TNPO3 serves as a protein decoy to competitively interact with IGF2BP3, and the stabilization role of IGF2BP3 on c-MYC mRNA is weakened, leading to inhibition of metastasis in gastric cancer 183.

The m^6^A modification is established by m^6^A methyltransferases (also known as m^6^A writer) 56. METTL3 and METTL14 are core subunits of the methyltransferase complex that efficiently catalyses m^6^A modification 184. Accumulating evidences in recent years demonstrate that METTL3, in most cases, plays as an oncogene in cancer 185. Importantly, in some instances, the function of METTL3 in cancer depends on m^6^A readers, such as YTHDF2 186. In gastric cancer, higher expression of METTL3 is associated with poor prognosis, and mechanistically, METTL3 mediates the m^6^A modification of HDGF mRNA in a manner with IGF2BP3-dependent HDGF mRNA stability 187. In addition to METTL3, another m^6^A writer, RBM15 regulates the m^6^A modification of its downstream target, TMBIM6, and enhances TMBIM6 mRNA stability through IGF2BP3-dependent way, and thereby facilitating progression of laryngeal squamous cell carcinoma 188. Interestingly, cross-talk among m^6^A writers, erasers, readers maintains the m^6^A level that regulates tumor growth and progression. For example, METTL14 and ALKBH5 (eraser) determine the m^6^A level of targets via controlling each other expression and by inhibiting YTHDF3 (reader) 189. In some cases, the m^6^A modification requires different readers to regulate their targets mRNA. For example, m^6^A-modified pyruvate dehydrogenase kinase 4 (PDK4) participates in glycolysis of cancer cells, and specifically, m^6^A-modified PDK4 regulates translation and mRNA stability via binding to YTHDF1 and IGF2BP3, respectively 121.

### IGF2BPs

**Figure 2:**
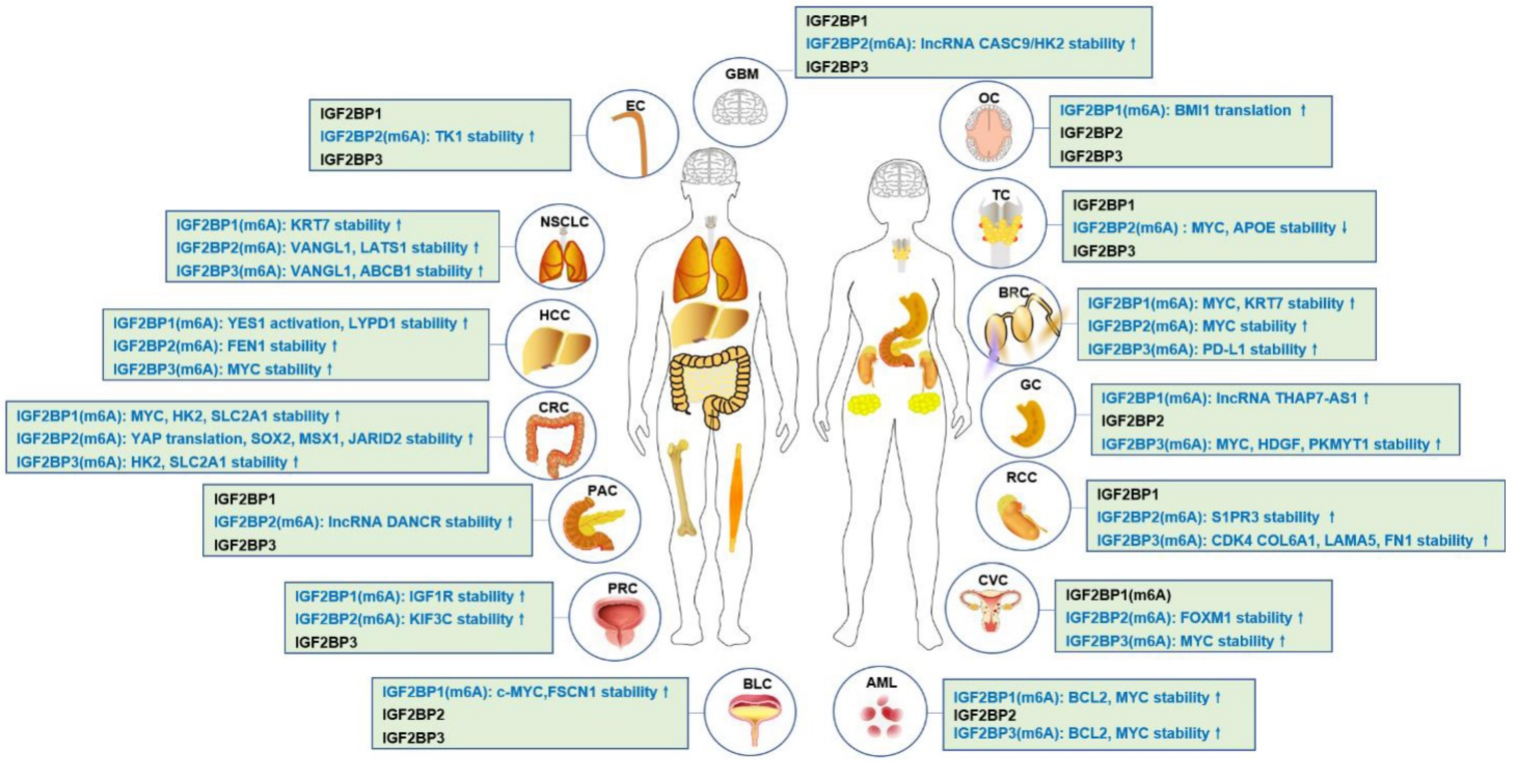
IGF2BPs-modified m^6^A in human cancers.

**Figure 3:**
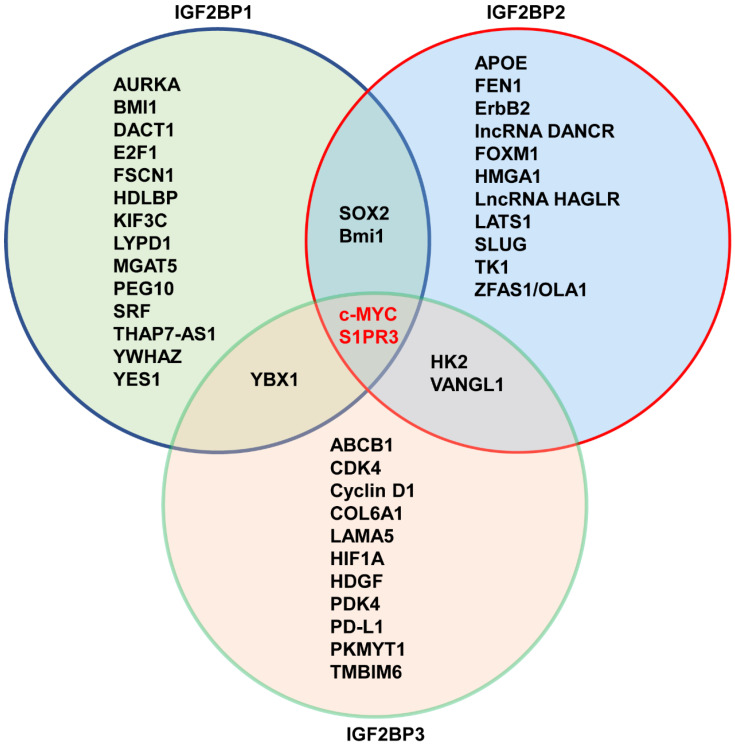
Overview of IGF2BPs-modified m^6^A on their downstream targets.

In 2018, IGF2BPs family was first identified as new m^6^A reader that has unique KH domains different from classical YTH domains 11. IGF2BPs promote the stability and storage of c-MYC, and the oncogenic role of IGF2BPs depends on their function as m^6^A readers 11. In renal cell cancer, transcription factor early growth response 2 (EGR2) increases the expressions of IGF2BPs, and IGF2BPs enhance the stability of sphingosine-1-phosphate receptor 3 (S1PR3) mRNA in m^6^A-dependent manner, and S1PR3 drives tumorigenesis and metastasis 190. In acute myeloid leukemia (AML), RNA-binding protein YBX1 is required for survival of AML, and mechanistically, YBX1 can cooperate with IGF2BP1 and IGF2BP3 to increase the stability of c-MYC and BCL2 mRNA in an m^6^A dependent manner 191. In hepatocellular carcinoma (HCC), the cancer-testis lncRNA-CTHCC promotes HCC growth and metastasis, and mechanistically, lncRNA-CTHCC is modified by m^6^A methylation with METTL3 and IGF2BP1/IGF2BP3 to maintain its stability and increase its expression 192. In colorectal cancer, METTL3 promotes glycolysis metabolism to drive tumorigenesis, mechanistically, METTL3 mediates m^6^A modification to enhance the expressions of HK2 and SLC2A1 through IGF2BP2/3-dependent mRNA stability function 193. In lung cancer, IGF2BPs, in particular, the IGF2BP2/3 increase the mRNA stability of VANGL1, and VANGL1 is associated with radio-resistance 194. Since IGF2BPs are oncofetal, interestingly, degradation of IGF2BPs can be used for cancer treatment. For example, tumor suppressor gene, circNDUFB2 interacts with the KH domains of IGF2BP1/2/3 in an m^6^A-dependent manner, and facilitates ubiquitination and degradation of IGF2BPs, thus leading to inhibition of tumor growth of lung cancer 195. The detail functions of IGF2BPs- modified m^6^A in cancer is shown in **Table 1 and Figure 2, Figure 3**.

## Conclusion and perspectives

IGF2BPs-mediated m^6^A modification that controls mRNA fate is emerging as a rising star in cancer through more mechanistic analyses since 2018. However, the oncogenic role of IGF2BPs in stabilizing theirs targets, such as c-MYC, has garnered interest in developing small-molecule inhibitors targeting IGF2BPs before 2018. As a result, a novel IGF2BP1 inhibitor, BTYNB, was identified to inhibit IGF2BP1 and destabilize c-MYC, providing a therapeutic option for cancer treatment 199. Besides IGF2BPs, several small-molecule inhibitors targeting other m^6^A modification proteins (writers, erasers) are discovered using high-throughput screening 200. Nevertheless, it is possible that inhibition of IGF2BPs may lead to feedback activation of other readers (such as YTHDF1), inevitably developing drug resistance.

IGF2BPs participate in posttranscriptional RNA processing, such as RNA splicing, translation, stability and decay, and in most cases, IGF2BPs stabilize their targets in m^6^A-dependent manner. Nevertheless, many questions remain to be resolved. (i) If IGF2BPs stabilize their downstream targets in an m^6^A-independent way, the detail mechanisms are what? (ii) Whether IGF2BPs increase the stability of targets mRNA through m^6^A modification before IGF2BPs are identified as new m^6^A readers? (iii) Do IGF2BPs and other m^6^A readers compete for the same targets mRNA, such as c-MYC? (ⅳ) How m^6^A writers (such as METTL3) and readers (IGF2BPs) cooperate through the “writers-readers system”? Thus, future structural studies are strongly warranted to understand how IGF2BPs binds to their targets, such as c-MYC, and investigate other KH domains-containing proteins as potential m^6^A readers.

## Figures and Tables

**Table 1:** The functions of IGF2BPs as m^6^A readers in cancer

Upstream target	m^6^A reader	Function	Cancer type	Reference
	IGF2BPs	Enhances c-MYC mRNA stability and translation	Pan-cancer	11
	IGF2BPs	Stabilizes VANGL1	Lung cancer	194
	IGF2BPs	Stabilizes HK2 and SLC2A1	Colorectal cancer	193
circNDUFB2	IGF2BPs	Enhances IGF2BPs degradation	Lung cancer	195
EGR2	IGF2BPs	Stabilizes S1PR3 mRNA	Renal cell carcinoma	190
YBX1	IGF2BPs	Stabilizes BCL2, c-MYC mRNA	Myeloid leukemia	191
	IGF2BPs	Stabilizes lncRNA-CTHCC	Hepatocellular carcinoma	192
lncRNA KB-1980E6.3	IGF2BP1	Stabilizes c-MYC	Breast cancer	137
LINC00266-1	IGF2BP1	Stabilizes c-MYC	Colorectal cancer	139
CircPTPRA	IGF2BP1	Stabilizes c-MYC and FSCN1	Bladder cancer	105
	IGF2BP1	Stabilizes PEG10	Endometrial cancer	140
	IGF2BP1	Promotes SRF expression	Pan-cancer	145
	IGF2BP1	Stabilizes MGAT5	Liver cancer	144
	IGF2BP1	Stabilizes AURKA, HDLBP and YWHAZ	Pan-cancer	196
PADI2	IGF2BP1	Stabilizes SOX2	Endometrial cancer	141
	IGF2BP1	Controls E2F1 turnover	Pan-cancer	87
RBM15	IGF2BP1	Promotes post-transcriptional activation of YES1	HCC	143
METTL3	IGF2BP1	Stabilizes KIF3C	Prostate cancer	147
METTL3	IGF2BP1	Stabilizes lncRNA THAP7-AS1	Gastric cancer	135
ALKBH5	IGF2BP1	Stabilizes LYPD1	HCC	142
METTL3	IGF2BP1	Promotes BMI1 translation	Squamous Cell Carcinoma	146
FTO	IGF2BP1	Reduce mRNA stability of DACT1	Osteosarcoma	149
	IGF2BP2	Reduces LncRNA HAGLR	Thyroid cancer	197
	IGF2BP2	Stabilizes FEN1 mRNA	HCC	92
	IGF2BP2	Promote YAP translation, and activates ErbB2	Colorectal cancer	163
	IGF2BP2	Stabilizes lncRNA DANCR	Pancreatic cancer	155
CircARHGAP12	IGF2BP2	Stabilizes FOXM1 mRNA	Cervical cancer	161
HCG11	IGF2BP2	Stabilizes LATS1 mRNA	Lung cancer	198
lncRNA CCAT2	IGF2BP2	Stabilizes TK1 mRNA	Esophageal squamous cell carcinoma	152
LINC01021	IGF2BP2	Enhances mRNA stability of MSX1 and JARID2	Colorectal cancer	159
	IGF2BP2	Stabilizes lncRNA CASC9/HK2 mRNA	Glioblastoma	154
	IGF2BP2	Stabilizes the ZFAS1/OLA1 axis	Colorectal cancer	158
METTL3	IGF2BP2	Prevents SOX2 mRNA degradation	Colorectal cancer	164
	IGF2BP2	Promotes Slug mRNA stability	Head and neck squamous carcinoma	168
FTO	IGF2BP2	Reduces APOE mRNA stability	Thyroid cancer	167
HPV E6/E7	IGF2BP2	Stabilize MYC expression	Cervical cancer	169
miR-204	IGF2BP2	Enhances c-MYC expression	Thyroid Cancer	156
LINC00460	IGF2BP2	Stabilizes HMGA1 mRNA	Colorectal cancer	150
	IGF2BP3	Stabilizes ABCB1 mRNA	Chemoresistance	178
lncRNA (DMDRMR)	IGF2BP3	Stabilizes CDK4 COL6A1, LAMA5, FN1	Renal cell carcinoma	111
	IGF2BP3	Stabilizes HIF1A	Gastric cancer	177
ALKBH5	IGF2BP3	Stabilize mRNA stability of PKMYT1	Gastric cancer	179
METTL3	IGF2BP3	Promotes PD-L1 mRNA activation	Breast cancer	180
MYC	IGF2BP3	Increases mRNA stability of KPNA2	Nasopharyngeal carcinoma	181
RBM15	IGF2BP3	Stabilizes TMBIM6	Laryngeal squamous cell carcinoma	188
	IGF2BP3	Reduces Cyclin D1 mRNA stability	Colon cancer	176
	IGF2BP3	Stabilizes HDGF	Gastric cancer	187
	IGF2BP3	Promotes translation and stability of PDK4	Pan-cancer	121

## Abbreviations

IGF2BPs: insulin-like growth factor-2 mRNA-binding proteins
RBPs: RNA-binding proteins
m^6^A: N6-methyladenosine
KH: K-homologous
RRMs: RNA recognition motifs
SAM: s-adenosylmethionine
METTL3: methyltransferase-like protein 3
MAC: m^6^A-METTL complex
MACOM: m6A-METTL associated complex
WTAP: wilms tumor 1 associated protein
VIRMA: vir-like m^6^A methyltransferase-associated
RBM15: RNA-binding motif 15
ZC3H13: zinc-finger CCCH-type-containing 13
CBLL1: Cbl proto oncogene-like protein 1
FTO: fat mass and obesity-associated protein
ALKBH5: α-ketoglutarate-dependent dioxygenase alk B homolog 5
m^6^Am: N6,2′-O-dimethyladenosine
YTHDFs: YT521-B homology domain family proteins
YTHDC1: YT521-B homology domain containing 1
eIF3: eukaryotic translation initiation factor 3
HNRNPs: heterogeneous nuclear ribonucleoproteins
Prrc2a: Proline rich coiled-coil 2 A
CNBP: cellular nucleic acid binding protein
SRSF3: serine and arginine-rich splicing factor 3
BCSC: stemness of breast cancer stem cells
PABPC1: polyadenylate-binding protein 1
PADI2: peptidylarginine deiminase II
LYPD1: LY6/PLAUR Domain Containing 1
HMGA1: high-mobility group AT-hook 1
TK1: thymidine kinase 1
IGF1R: insulin-like growth factor1 receptor
HK2: hexokinase 2
FOXM1: forkhead box M1
FEN1: flap endonuclease-1
AURKA: aurora kinase A
C/EBPs: CCAAT/enhancer binding proteins
VEGF: vascular endothelial growth factor
HIF1A: hypoxia inducible factor-1a
ABCB1: ATP-binding cassette transporters subfamily B member 1
PDK4: pyruvate dehydrogenase kinase 4
EGR2: early growth response 2
